# Visualization of Src and FAK Activity during the Differentiation Process from HMSCs to Osteoblasts

**DOI:** 10.1371/journal.pone.0042709

**Published:** 2012-08-10

**Authors:** Xiaoling Liao, Shaoying Lu, Yue Zhuo, Christina Winter, Wenfeng Xu, Yingxiao Wang

**Affiliations:** 1 Biomaterials and Live Cell Imaging Institute, School of Metallurgy and Materials Engineering, Chongqing University of Science and technology, Chongqing, People’s Republic of China; 2 Department of Bioengineering, University of Illinois, Urbana-Champaign, Urbana, Illinois, United States of America; 3 Beckman Institute for Advanced Science and Technology, Center for Biophysics and Computational Biology, Institute for Genomic Biology, Department of Integrative and Molecular Physiology, University of Illinois, Urbana-Champaign, Urbana, Illinois, United States of America; National Centre for Scientific Research, ‘Demokritos’, Greece

## Abstract

Non-receptor protein kinases FAK and Src play crucial roles in regulating cellular adhesions, growth, migration and differentiation. However, it remains unclear how the activity of FAK and Src is regulated during the differentiation process from mesenchymal stem cells (MSCs) to bone cells. In this study, we used genetically encoded FAK and Src biosensors based on fluorescence resonance energy transfer (FRET) to monitor the FAK and Src activity in live cells during the differentiation process. The results revealed that the FAK activity increased after the induction of differentiation, which peaked around 20–27 days after induction. Meanwhile, the Src activity decreased continuously for 27 days after induction. Therefore, the results showed significant and differential changes of FAK and Src activity upon induction. This opposite trend between FAK and Src activation suggests novel and un-coupled Src/FAK functions during the osteoblastic differentiation process. These results should provide important information for the biochemical signals during the differentiation process of stem cells toward bone cells, which will advance our understanding of bone repair and tissue engineering.

## Introduction

**Figure 1:**
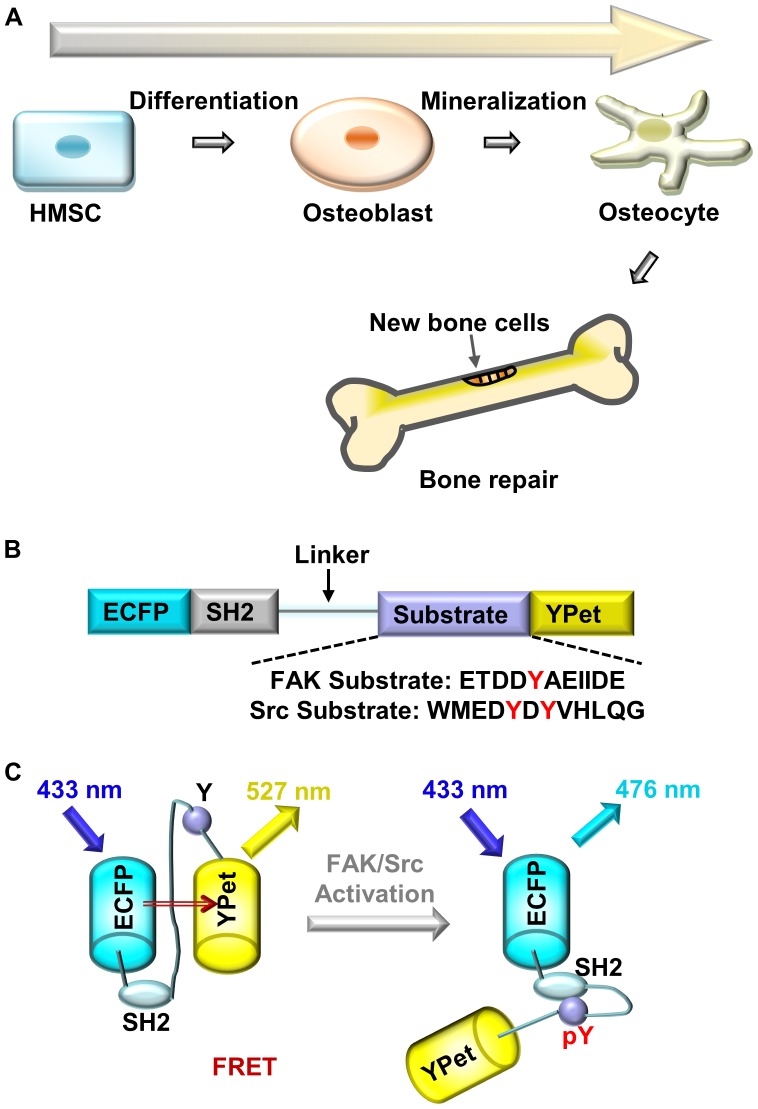
The schematics of HMSC-osteoblastic differentiation and the FRET biosensors. (**A**) The HMSCs differentiate into osteocytes through osteoblasts. (**B**) The schematic design of FRET biosensors (FAK/Src); (**C**) The activation mechanism of the FRET biosensors (FAK/Src), the letter “Y” represents a tyrosine site on the specific substrate peptide, and “pY” in red color represents a phosphorylated tyrosine.

Human mesenchymal stem cells (HMSCs) have excellent immunosuppression and anti-inflammatory properties. They are capable of self-renewal and differentiation into multiple tissues, including bone, cartilage, muscle, fat, skin and connective tissues [1], [2]. Therefore, HMSCs can be used in bone, cartilage, and ligament repair, and potentially in gene-therapy-based bone tissue engineering [3]–[6]. In bone tissue engineering, HMSCs are often cultured *in vitro* and allowed to expand and differentiate on a biocompatible scaffold, which mimics naturally occurring tissues. The cell-seeded scaffold can later be implanted into the patients (Fig. 1A) [3], [7], [8]. The successful expansion and differentiation of HMSCs on the scaffold therefore constitutes a crucial step in bone tissue engineering.

The differentiation of HMSCs is modulated by complex and constant “crosstalk” among different intracellular and extracellular signals [9], [10]. The importance of these regulatory signaling pathways in the remolding and maintenance of the skeleton is widely accepted. However, the mechanisms involved in HMSC differentiation are just starting to unfold [6], [11], [12]. Therefore, monitoring and understanding the molecular events triggered during the osteogenesis process of HMSCs will provide important insights for the development of innovative approaches for effective HMSC-based therapeutics.

The signaling pathways regulating the stem cell differentiation involve numerous cytokines, growth factors, hormones, and extracellular matrix (ECM) and mechanical environment [12]–[15]. Among these, several signaling pathways are recognized as regulating the osteogenesis process of HMSCs, such as those regulated by bone morphogenetic proteins (BMPs), and fibroblastic growth factors (FGFs) [13], [14]. BMPs are secreted proteins that belong to the transforming growth factor-β (TGF-β) super family. They can up-regulate intracellular signals such as the canonical mothers against decapentaplegic (Smad) and p38 mitogen activated protein kinase (MAPK), and subsequently the expression of the runt-related transcription factor 2 (Runx2). The BMPs can also up-regulate the expression of the zinc finger-containing transcription factor, osterix, either dependent or independent of Runx2. The over-expression of Runx2 and osterix can then cooperate and lead to osteogenesis and bone formation [14], [16]. Indeed, it has been shown that the bone induction reagent containing dexamethasone and β-sodium glycerophosphate can up-regulate BMP expression and subsequently ALP activity and Runx2 expression [17], [18]. FGFs have also been shown to serve as a strong regulator for both osteogenesis and adipogenesis in the presence of their respective differentiation factors [14]. In addition to these soluble biochemical stimuli, extracellular matrix proteins and mechanical forces can control the differentiation of HMSCs via mechanosensing [12].

However, many details, especially the crosstalk among these signaling pathways, remain unclear due to the lack of explicit information. For instance, the details of how the growth factors and ECM-integrin signaling pathways crosstalk and co-regulate the non-receptor protein kinases, such as focal adhesion kinase (FAK) and Src during stem cell differentiation, remain unclear. FAK is considered to be one of the main mechanotransduction signaling proteins at the cell-matrix adhesion sites. FAK and Src are known to form complexes and cooperate at the focal adhesion sites to promote cell migration, proliferation, survival and differentiation [19]–[24]. ECM contacts can signal through FAK to activate MAPK and stimulate the subsequent phosphorylation of Runx2 and osteogenesis [25], [26]. It has been reported that the bone induction reagent can transiently increase the level of FAK tyrosine phosphorylation, but not expression [27]. On the other hand, Src is also an important signaling protein for maintaining bone homeostasis. It has been found that inhibiting Src activity can enhance the differentiation of osteoblasts and contribute to increasing bone mass [11], [28], [29]. Therefore, FAK and Src can regulate a cell’s fate during osteogenesis either cooperatively or independently. However, the long-term behavior of FAK and Src activities in stem cells during the differentiation process toward osteoblasts (Figure 1A) has not been investigated and remains obscure.

Recently, fluorescence resonant energy transfer (FRET)-based biosensors have been widely used to evaluate specific molecular activities in live cells [30], [31]. These biosensors utilize the principle of energy transfer between the donor and acceptor fluorescence proteins to detect the change in their distance or orientation caused by the molecular activity of a target protein. Based on this principle, Src and FAK FRET biosensors were previously developed and characterized by our group [20], [32], [33]. The current Src and FAK biosensors utilize similar design strategies, both containing an enhanced cyan fluorescent protein (ECFP as the FRET donor), a Src SH2 domain, a flexible linker, a specific tyrosine-containing substrate peptide, and a variant of the yellow fluorescent protein (YPet as the FRET acceptor, Fig. 1B) [20], [33]. The substrate sequence of the FAK biosensor is derived from the FAK auto-phosphorylation site tyrosine-397 [20], while the substrate peptide of the Src biosensor from a primary *in vivo* Src substrate p130cas [32]. Active Src or FAK can promote tyrosine phosphorylation on the substrate peptide of the specific biosensor. The phosphorylated tyrosine subsequently binds to the intramolecular SH2 domain, which causes a conformational change and a decrease of FRET efficiency between the donor and acceptor (Fig. 1C). The decrease of FRET efficiency can be visualized as an increase of the ratio of the donor/acceptor emission intensity, namely the FRET ratio, when the donor FP is excited. As a result, the Src or FAK activity can be represented by the FRET ratio between the donor and acceptor emissions [20], [32], [33].

FAK and Src biosensors have been used successfully to monitor FAK and Src activity in live cells [20], [32]–[34]. In the current study, we visualized the FAK and Src activities in HMSCs using these biosensors during the differentiation process induced by bone induction reagents [35], [36]. Changes in FAK and Src activity were detected during the differentiation process from HMSCs to osteoblasts (at 0, 8, 15, 20 and 27 days) after the application of these bone induction reagents.

## Results

### FAK Activity Increased during Osteogenic Differentiation

**Figure 2:**
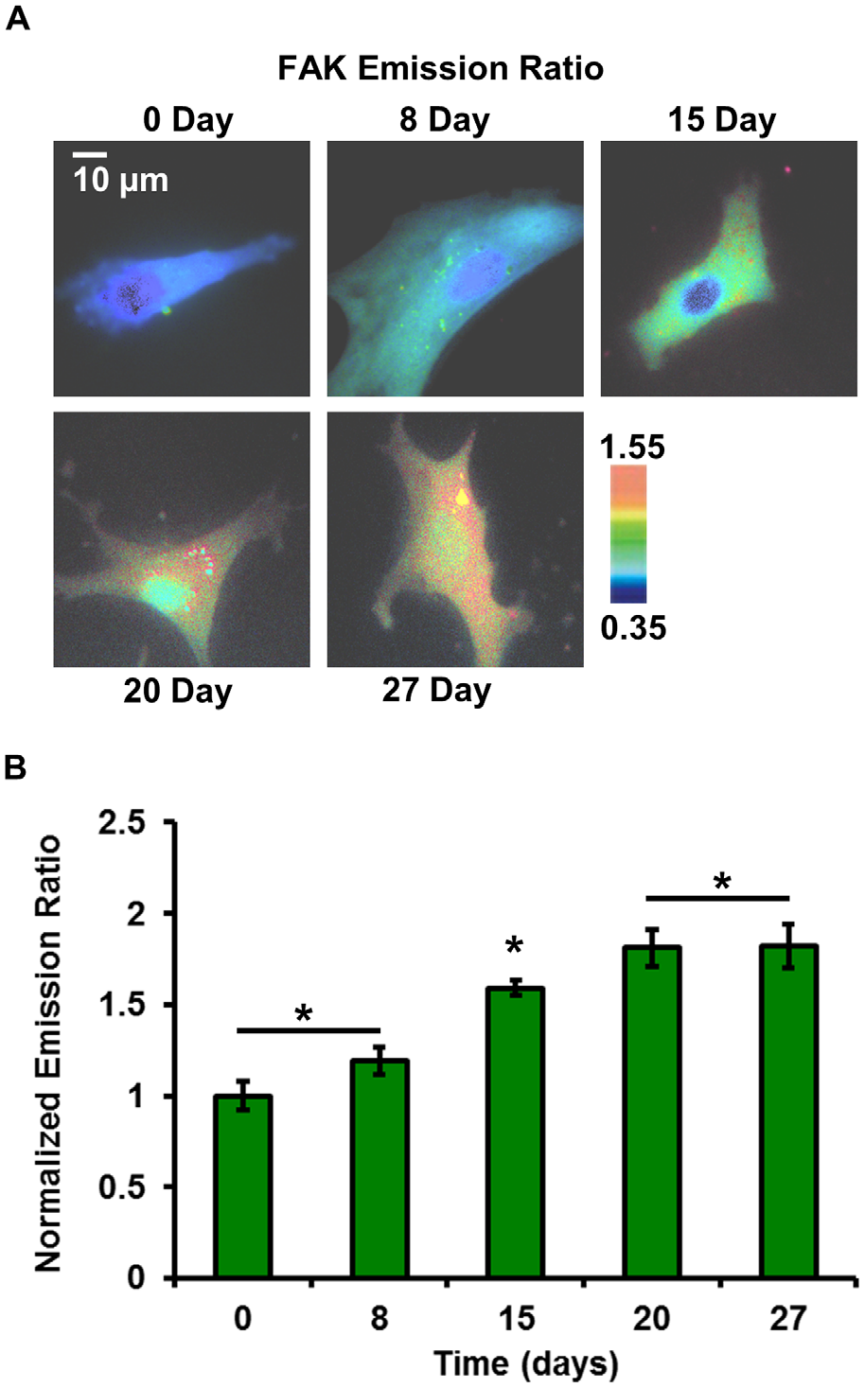
The FAK kinase activity increased during the differentiation process from HMSCs to osteoblasts. (**A**) The emission ratio images of the FAK biosensor from day 0 to day 27 during the differentiation process in live cells. (**B**) Normalized emission ratio of the FAK biosensor during the differentiation process in live cells (mean ±S.D., n = 12). The symbol ‘*’ denotes significant difference from other groups by the Bonferroni multiple comparison test in MATLAB, p-value<0.05.

We examined the changes in FAK signaling events in live cells during the differentiation process from HMSCs, at 0, 8, 15, 20 and 27 days after induction (Fig. 1A). FAK biosensors were transfected into the cells to visualize the spatiotemporal dynamics of FAK activation in HMSCs during the osteoblastic differentiation process. Figure 2A shows the emission ratio images of FAK biosensors at various time points during the differentiation process in live stem cells. From the emission ratio images, we can observe that there is a time-dependent increase of FAK activity across the whole cell, except for the nucleus. The sensitivity and specificity of this FAK biosensor has previously been extensively characterized *in vitro* and in FAK knockout cells co-expressing the biosensor and various FAK mutants. It has been confirmed that the observed increase in FRET ratio was due to the phosphorylation of the biosensor via active FAK kinase [20].

To further study the increasing trend of FAK activity, we quantitatively measured the FAK activity represented by the biosensor FRET ratio among the group of cells examined. As shown in Figure 2B, the average FAK activity, normalized to its value at day 0, increased in time (day 0: 1.0±0.1264; day 8: 1.1899±0.1183; day 15: 1.5904±0.0671, mean ±S.D.), and peaked after 20 days with about 1.8 fold induction compared to day 0. In comparison to HMSCs before differentiation, FAK activity showed a significantly higher value at these later time points at a relatively stable level (day 20: 1.8097±0.1558; day 27: 1.8213±0.1781, mean ±S.D.). These results show that FAK kinase activity increased steadily during the osteogenic differentiation process to reach a plateau after 20 days. For statistical comparison among multiple groups, the Bonferroni multiple-comparison test in MATLAB (by the *multcompare* function) was used.

### Src Activity Decreased during Osteogenic Differentiation

**Figure 3:**
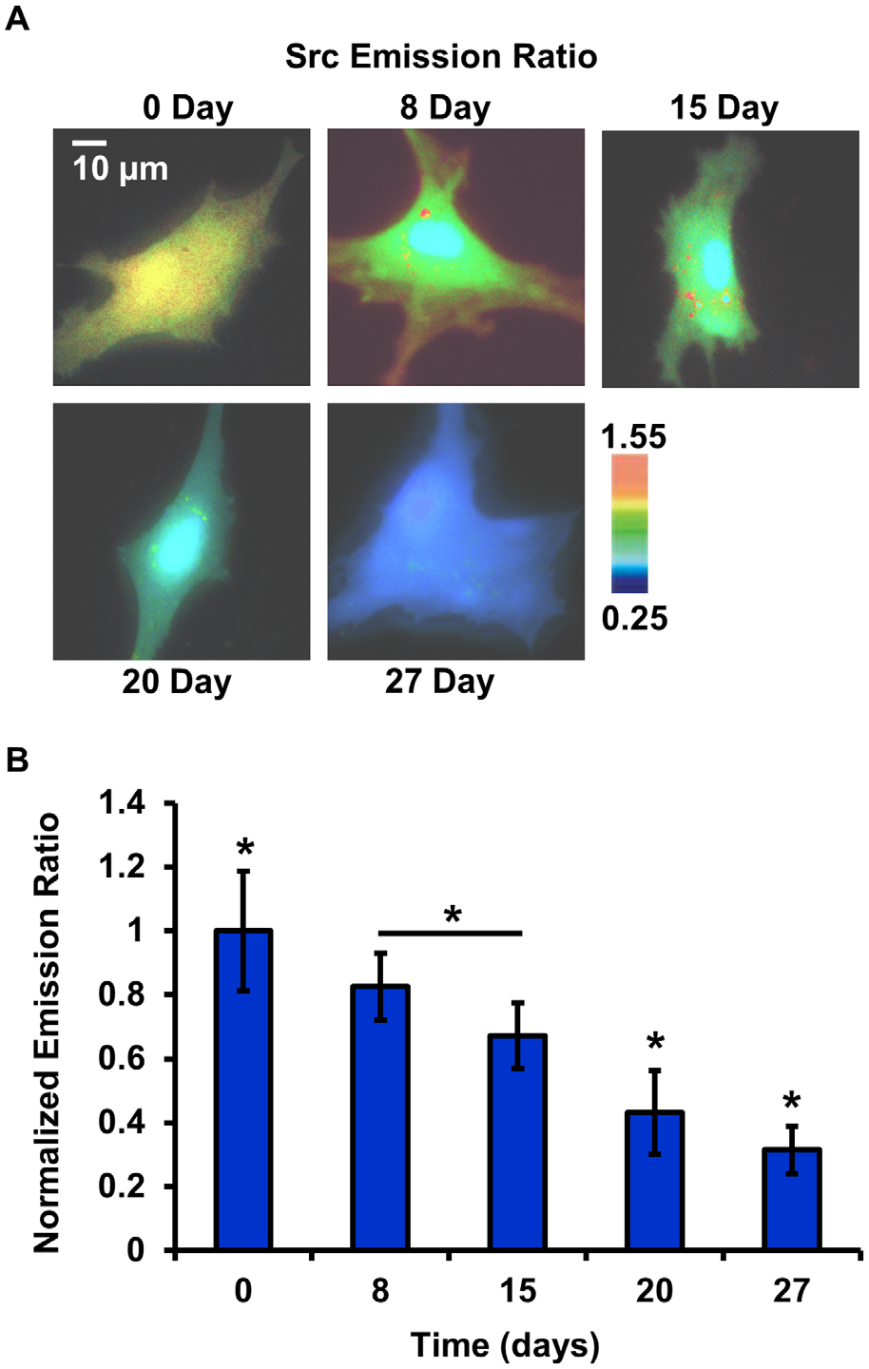
The Src kinase activity decreased during the differentiation process in live cells. (**A**) The emission ratio images of the Src biosensor from day 0 to day 27 during the differentiation process from HMSCs to osteoblasts. (**B**) Normalized emission ratio of the Src biosensor during the differentiation process in live cells (mean ±S.D., n = 12). The symbol ‘*’ denotes significant difference from other groups by the Bonferroni multiple comparison test in MATLAB, p-value<0.05.

Similarly, Src signaling events were examined during the differentiation process from HMSCs to osteoblasts (in the same time scale as that of images on FAK activity). The spatiotemporal activation of Src was similarly monitored by the FRET ratio during the differentiation process. As shown in Figure 3A, the Src biosensor is mainly distributed in the cytosol of the cells. However, the Src kinase activity, as indicated by the ECFP/FRET emission ratio of Src biosensors, decreased consistently during the differentiation time course, which is the opposite of FAK kinase activity. The sensitivity and specificity of the Src biosensor substrate sequence has previously been characterized *in vitro* and in mammalian cells [32].

Again, the decreasing trend of Src activities observed on single cells was further confirmed with the statistical results among multiple cells, as shown in Figure 3B. The normalized Src activity continuously decreased along the time course of differentiation (day 0: 1.0±0.1523; day 8: 0.8258±0.0847; day 15: 0.6725±0.0837, mean ±S.D., day 20: 0.4327±0.1063; day 27: 0.3157±0.0601, mean ±S.D.), resulting a 0.68-fold decrease from day 0 to day 27.

### The Change of ALP Activity

**Figure 4:**
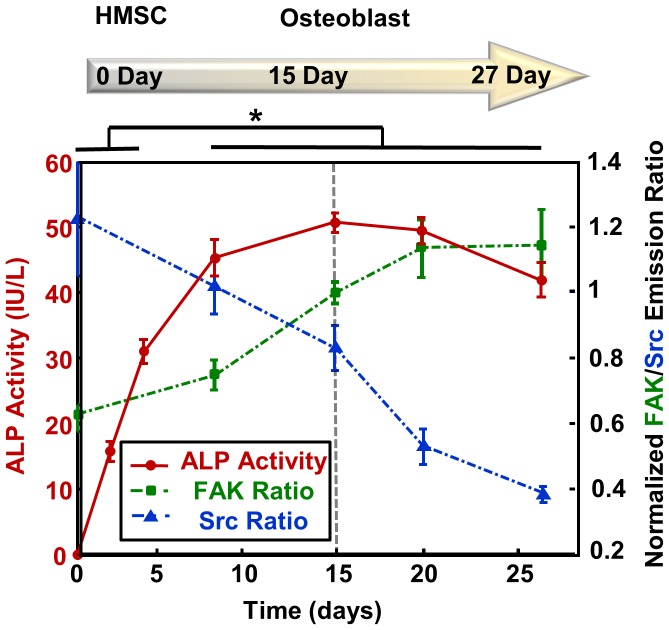
The biphasic trend of ALP (alkaline phosphatase) expression and monotonic change of FAK/Src kinase activities during the bone cell differentiation process, measured by an ALP reagent set and a UV spectrophotometer (mean ±S.D., measured at 405 nm). The symbol ‘*’ denotes significant difference in ALP expression by t-test, p-value<0.05.

To confirm the status of HMSCs when differentiating into osteoblasts in our experiment, we examined the change of alkaline phosphatase (ALP) activity during this process. ALP is an important functional marker and sensitive target to reflect the osteoblastic activity [37]. ALP has two functions in the osteoblast differentiation process: 1) transforming organophosphorus to inorganic phosphorus in osteoblasts, which is needed for the mineralization of osteoblasts; 2) promoting the dephosphorylation of corresponding substrates. The examination is performed with the ALP reagent set and UV spectrophotometer (measured at 405 nm). As shown in Figure 4, in comparison to the monotonic trend of the changes in FAK and Src activities, there are two phases in ALP activity changes. Phase I (0–15 days, ascending phase): no obvious ALP activity was shown in HMSCs before differentiation (day 0: 0±0 IU/L, mean ±S.D.). After the differentiation procedure started, the ALP level started to increase (day 2: 15.62±1.523 IU/L; day 4: 30.89±1.861 IU/L; day 8: 45.2±2.862 IU/L) until it peaked around 15 days after induction (day 15: 50.6±1.497 IU/L). This trend of increasing ALP activity is consistent with previous observations on the differentiation procedure from HMSCs to osteoblasts [38]; Phase II (15–27 days, descending phase): the ALP activity started to decrease (day 20: 49.3±1.981 IU/L; day 27: 41.9±2.648 IU/L, day 32: 30.1±1.792 IU/L). This second phase suggests that the osteoblasts may start toward the maturation process [37]. As such, these results indicate that HMSCs successfully differentiated toward osteoblasts after the induction.

## Discussion

At the current stage, the advanced technology in tissue engineering involves multidisciplinary areas, such as material science, bioengineering, biology and medicine [2], [3]. Biocompatible scaffold is not only a simple material to support the stem cells for their differentiation into bone cells, but also an active reagent regulating the whole tissue-generating procedure including cell differentiation, migration, survival and proliferation. An ideal bone-repairing scaffold should mimic the natural bone tissue, which provides the cell-preferable structures and materials as well as the proper functional microenvironment to enhance sub-cellular activity [6], [12]. Therefore, understanding the mechanism of reagent-induced differentiation, especially the sub-cellular and molecular signaling pathways, is essential for tissue engineering and regenerative medicine.

Current evaluation methods for discovering the potential of tissue engineering biomaterials are largely limited to using biochemical assays on the cell lysate or immunostaining cells after fixation, which may result in the loss of information or introduction of artifacts [30], [31]. FRET-based biosensors have been widely used in live cells to monitor molecular activities or intermolecular interaction in real time [30], [39]. The ratio-metric measurement utilizes the ratio of the donor to acceptor fluorescence intensity to represent the target molecular activity. This measurement is independent of the heterogeneous biosensor expression level among various cells and the optical variability among different experiments. These genetically engineered biosensors also allow subcellular localization to cytosol, plasma membrane, or organelles, which can provide versatile measurement of subcellular molecular activities. Therefore, FRET biosensors can provide powerful tools in deciphering the molecular mechanisms governing the differentiation processes.

It has been reported that FAK can be activated during the osteogenesis process induced by shear stress or high stiffness of ECM substrates [19], [40], [41]. In addition, bone induction media containing dexamethasone and β-sodium glycerophosphate can transiently promote FAK tyrosine phosphorylation [17], [27], but its effect on FAK kinase activities was not clear. Our results showed that FAK was continuously activated for 20 days during differentiation induced by the bone induction reagents, highlighting the potential importance of FAK kinase activity when HMSCs differentiate toward osteoblasts. It is possible that FAK activity can enhance the cell-matrix contacts and promote the mechano-sensitivity of the cells toward the hard bone tissue. In fact, it has been demonstrated that FAK activity is higher in cells seeded on harder surfaces comparing to those on soft substrates [42].

Src is an oncogenic tyrosine kinase which also plays important roles in many cellular functions, including cell migration and differentiation [11], [24], [43]. It was not clear how Src activity changed during osteoblastic differentiation. Our results suggest that Src kinase activity decreased monotonically for 27 days during the differentiation process. This down-regulation of Src kinase activity may facilitate the osteogenesis process, since Src expression and activity have been found to inhibit the differentiation of HMSCs toward osteoblasts [28], [29]. This is also consistent with the non-migrative phenotype of the osteocytes at the maturation stage since Src family kinases have been known to promote cell migration and spreading [44], [45].

Although FAK and Src have been shown to cooperate at the focal adhesion sites, our results clearly indicate that their activities are distinctively regulated during HMSC differentiation into osteoblasts. This is consistent with recent findings that the majority of Src is not localized at focal adhesion sites as FAK is [46]. It is hence possible that only a small fraction of Src and FAK co-localize in cells, e.g. at focal adhesions. This is also supported by the observation that FAK activity resides mainly inside lipid rafts while Src activity is concentrated in non-raft regions at the plasma membrane [20], [47]. These different localizations of FAK and Src may result in their distinct regulation mechanisms during the differentiation process of HMSCs.

In summary, our results showed that FAK activity continuously increased while Src activity decreased when the HMSCs were induced to differentiate toward osteoblasts. This opposite trend between FAK and Src activation suggests novel and un-coupled Src/FAK functions during the osteoblastic differentiation process. This result on the regulation of FAK and Src activities is also consistent with the need for the stem cells to form more focal adhesions and became less motile on the substrate matrix during the osteogenic process. Further study on the signaling pathways connecting FAK/Src to gene regulation during HMSCs differentiation can greatly benefit the area of tissue engineering.

## Materials and Methods

### Cell Culture and Reagents

The HMSCs were purchased from ATCC. The cell culture reagents were obtained from Invitrogen. Cells were maintained in DMEM supplemented with 10% fetal bovine serum (FBS), sodium pyruvate (1 mM), penicillin (1 U/ml), and L-glutamine (2 mM). Cells were cultured in a humidified 5% CO_2_ (and 95% air) incubator at 37°C. The differentiation was stimulated with an induction reagent with dexamethasone (10^−8^ mol/L), vitamin D3 (50 mg/L), β-sodium glycerophosphate (10 mM) and ascorbate 2-phosphate (50 mg/L) [35], [36].

### Gene Constructions and DNA Plasmids

The FAK and Src biosensors were previously developed and characterized [20], [33]. As shown in Figure 1B, the Src/FAK biosensors were constructed by fusing the SH2 domain from c-Src, a flexible linker, and a specific substrate peptide between the N-terminus ECFP and the C-terminus YPet. The DNA encoding the Src/FAK biosensors were sub-cloned with the BamH1/EcoR1sites in pRSetB for the protein purification from *E-coli*, and in pcDNA3 plasmid for the expression and localization in the cytosol of mammalian cells [20], [33].

### Microscope and Image Acquisition

The cells were cultured in cover-glass-bottom dishes (Cell E&G) and kept in CO_2_-independent medium with 0.5% FBS (GIBCO BRL) at 37°C during imaging. Images were collected with a Nikon microscope with a charge-coupled device (CCD) camera. The FRET emission ratio images were generated by the MetaFluor 6.2 software (Universal Imaging), and then subjected to quantification and analysis by Excel (Microsoft).

### ALP Activity Assay

The induced HMSCs were harvested, followed by cell lysis with TritonX-100. The ALP activities were quantified by an ALP detection kit (Boshide company) [18]. The quantified results were normalized by the protein concentration loaded.
